# Complicated atrial myxoma with coro-cameral fistula arising from its feeding branch: a case report

**DOI:** 10.3389/fcvm.2024.1445366

**Published:** 2024-10-08

**Authors:** Jian-Qiang Li, Ping Dong, Qian-Li Wang, Xiao-Xia Li, Tu-Min Sha, Peng Zhang, Zhen-Qing Zhao, Chao-Liang Liu

**Affiliations:** ^1^ Department of Cardiac Surgery, Yantai Yuhuangding Hospital, Yantai, China; ^2^ Department of Hematology, Yantai Yuhuangding Hospital, Yantai, China; ^3^ Department of Intensive Care Unit, Yantai Yuhuangding Hospital, Yantai, China; ^4^ Department of Pathology, Yantai Yuhuangding Hospital, Yantai, China

**Keywords:** left atrial myxoma, coronary artery fistula, feeding artery rupture, cardiac tumors, atrial fibrillation

## Abstract

A 56-year-old man with a 5-year history of paroxysmal palpitations, which have worsened over the past year, was diagnosed with atrial fibrillation. During evaluation, transesophageal echocardiography revealed a left atrium (LA) tumoral mass attached to the atrial septal fossa ovale, with intra-tumoral blood flow and blood stream draining from the mass. Both coronary computed tomography angiography and coronary angiography demonstrated a coro-cameral fistula connection between the left circumflex artery (LCX) branch and the LA. In addition, they showed feeding arteries of the mass arising from the LCX. The patient underwent surgical resection of the LA mass and repair of the coronary artery fistula. Intraoperative exploration revealed a 1.7 cm × 1.0 cm jelly-like, brittle LA mass and confirmed a rupture of the supplying artery, leading to a coronary artery–left atrial fistula. Surgical ligation was executed to ensure complete sealing of the supplying coronary branch within the atrial septum. Histopathological examination confirmed the diagnosis of left atrial myxoma. The 6-month follow-up indicated no recurrence of the myxoma and restoration of sinus rhythm after radiofrequency ablation. In the literature, cases of a left circumflex artery branch–left atrial fistula due to rupture of the artery supplying a left atrial myxoma are rare.

## 1 Introduction

Primary cardiac tumors are exceedingly rare, representing a mere 0.03% of autopsy findings (1). Of these cases, an impressive 80% are benign, with myxomas accounting for nearly half of these benign tumors (2). Coronary artery fistulas (CAFs) are rare coronary anomalies characterized by abnormal connections between a coronary artery and a cardiac chamber or major vessels (3). CAFs can be congenital or acquired, with congenital types being the most common. Acquired CAFs can result from trauma, therapeutic procedures, or surgical interventions (4). To the best of our knowledge, the formation of a coronary-to-atrial fistula through a tumor has been rarely documented in the literature. Herein, we present a unique case of a left atrial myxoma leading to an acquired fistula between a branch of the left circumflex artery (LCX) and the left atrium (LA), caused by the rupture of the supplying artery within the myxomatous tissue.

## 2 Case report

**Figure 1:**
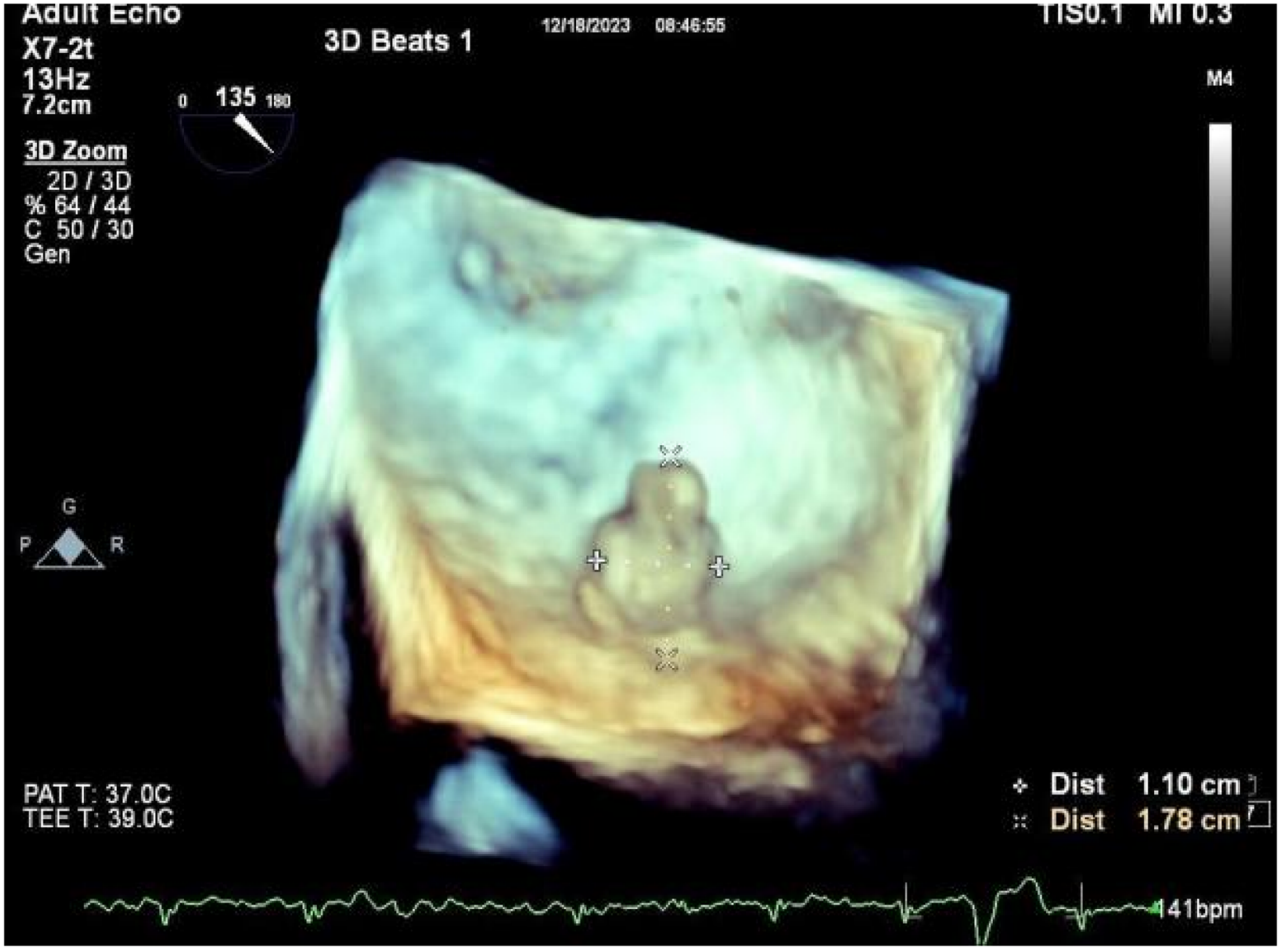
3D TEE revealed a 17.8 mm × 11 mm LA tumoral mass, attached to the atrial septal fossa ovale.

**Figure 2:**
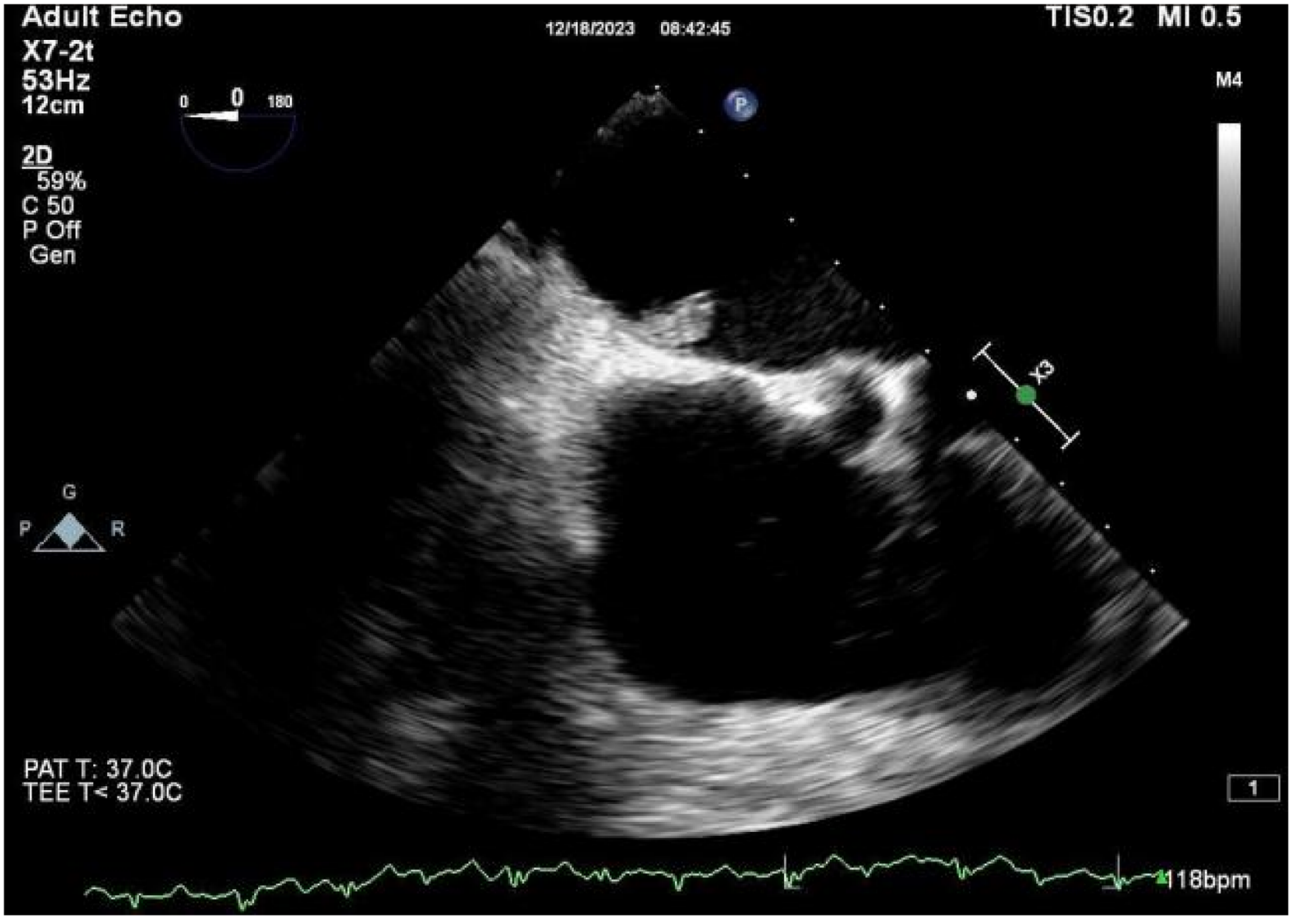
2D TEE revealed a mass with heterogeneous echogenicity attached to the atrial septal fossa ovale.

**Figure 3:**
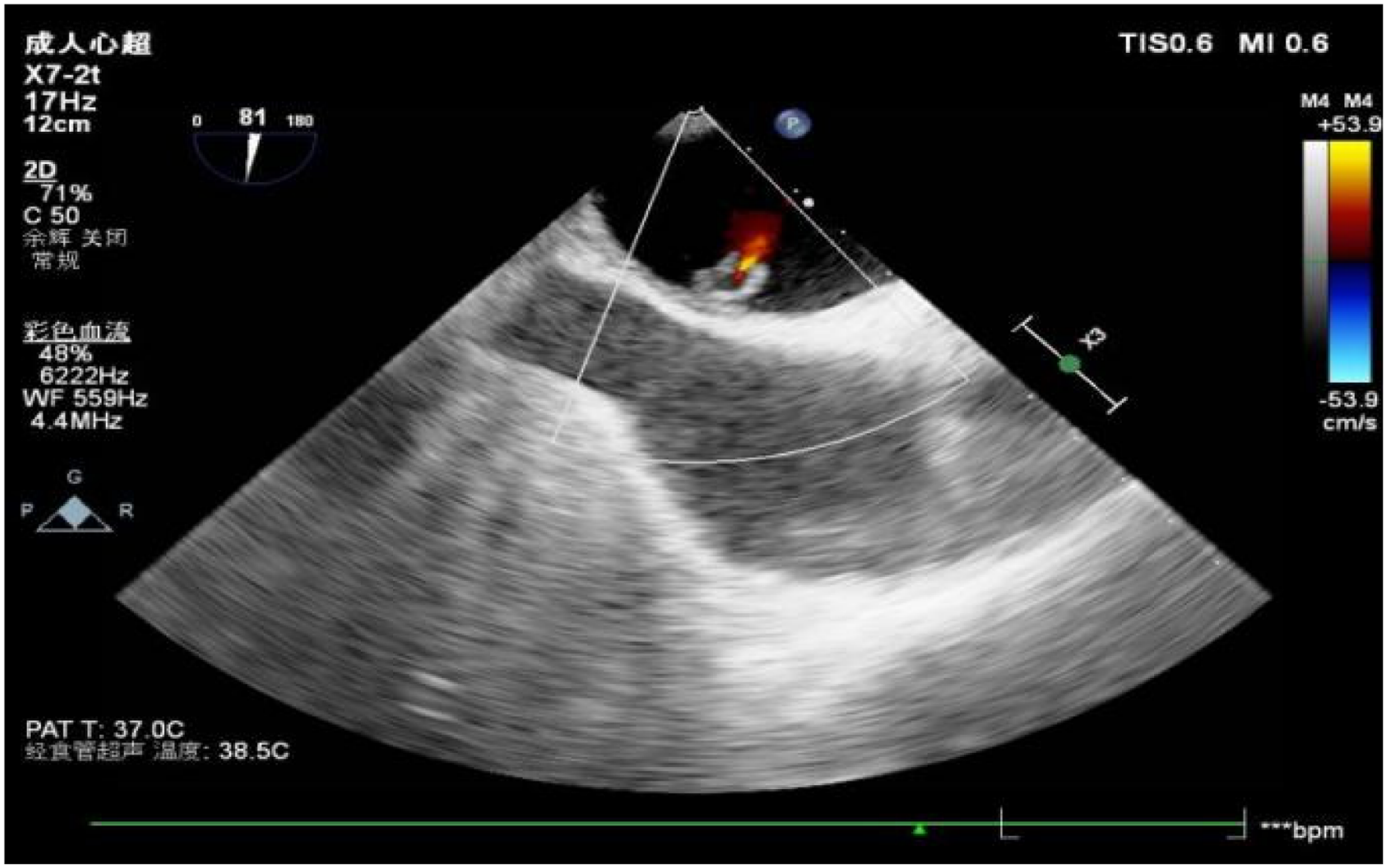
Color Doppler images showed intra-tumoral blood flow and blood stream draining from the mass.

**Figure 4:**
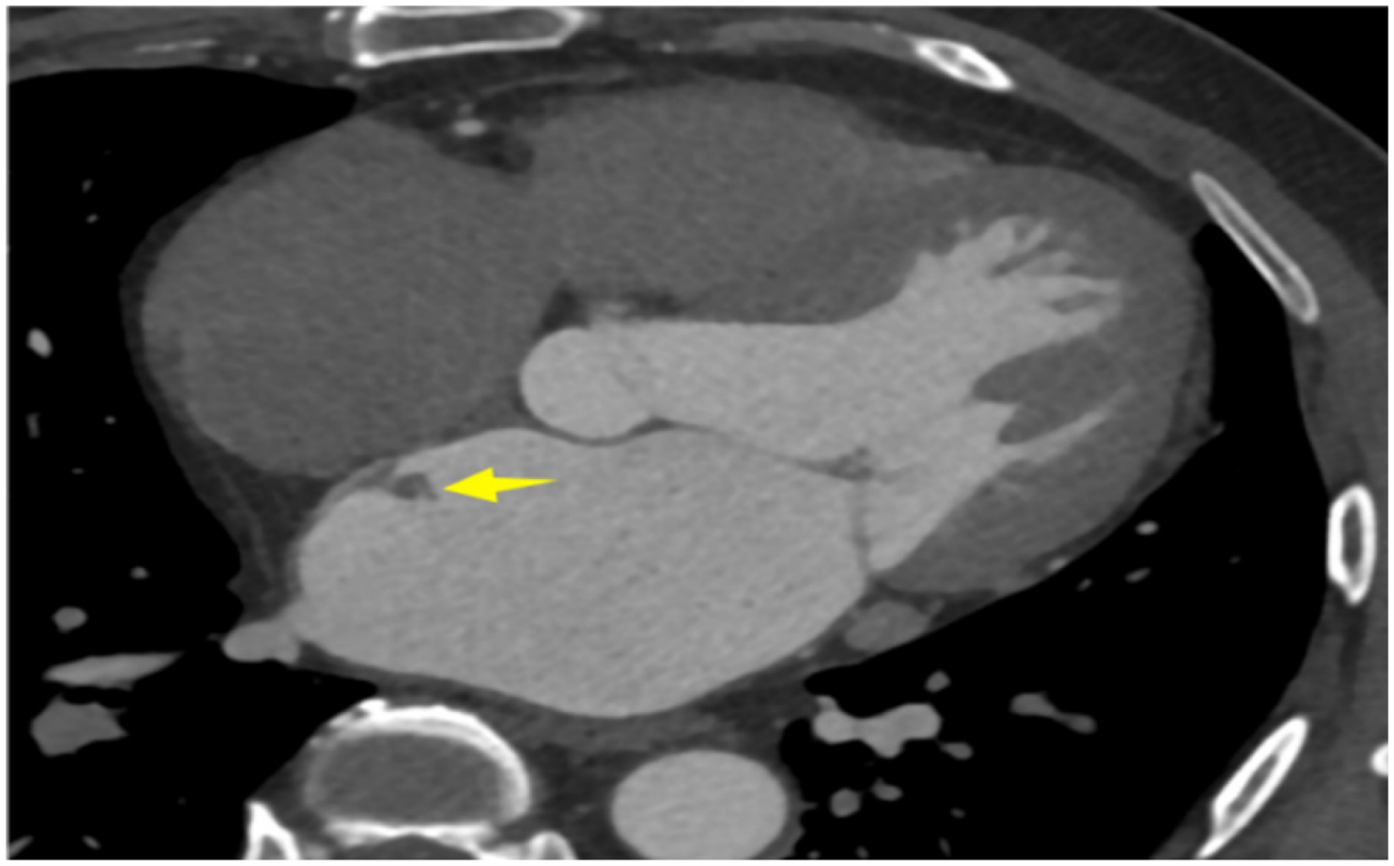
Left atrium CTA showed a mass in the left atrium arising from the atrial septal.

**Figure 5:**
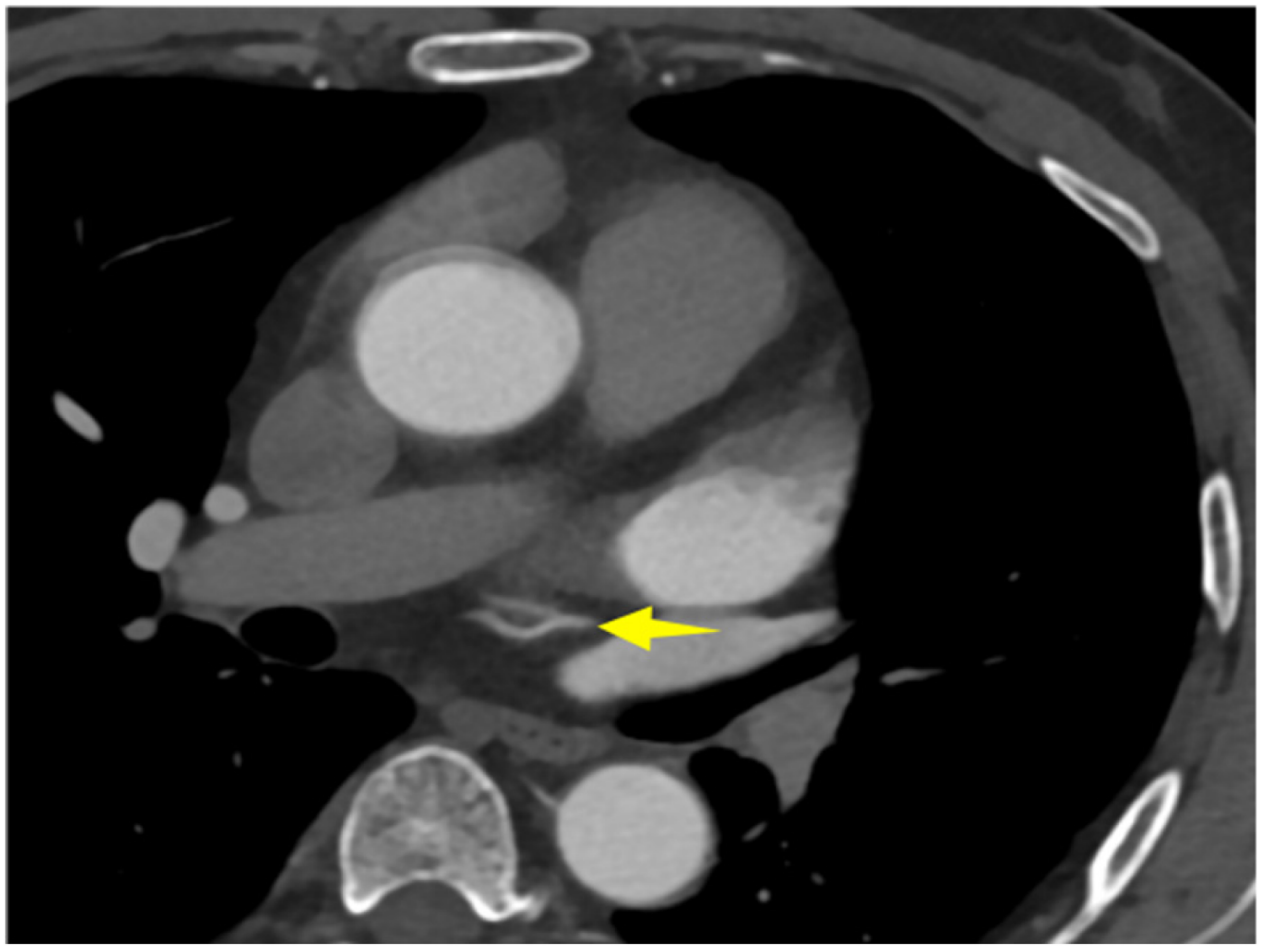
Coronary CTA showed a feeding artery of myxoma from LCX.

**Figure 6:**
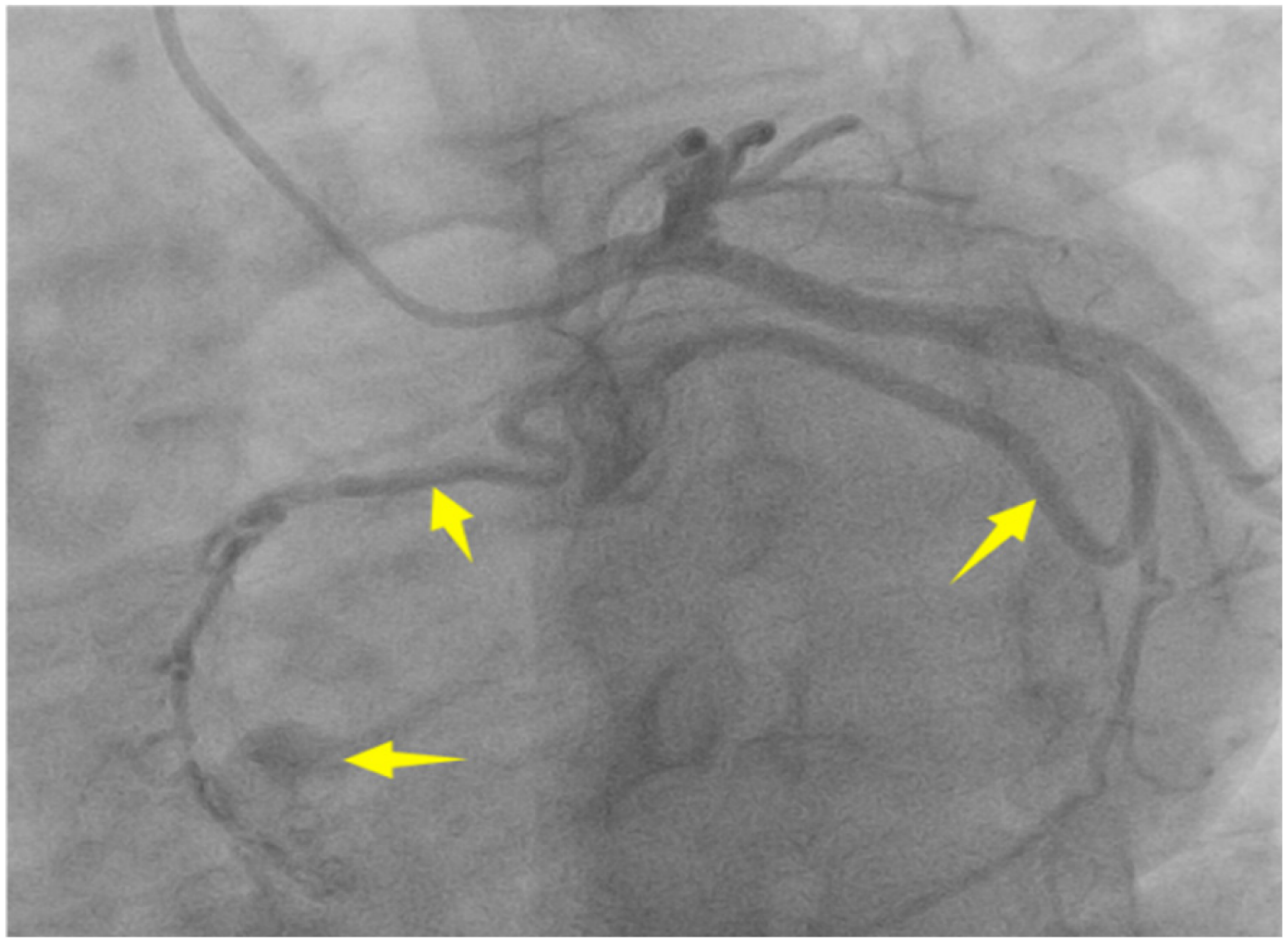
CAG revealed a coro-cameral fistulous connection between a branch of the LCX and the LA.

**Figure 7:**
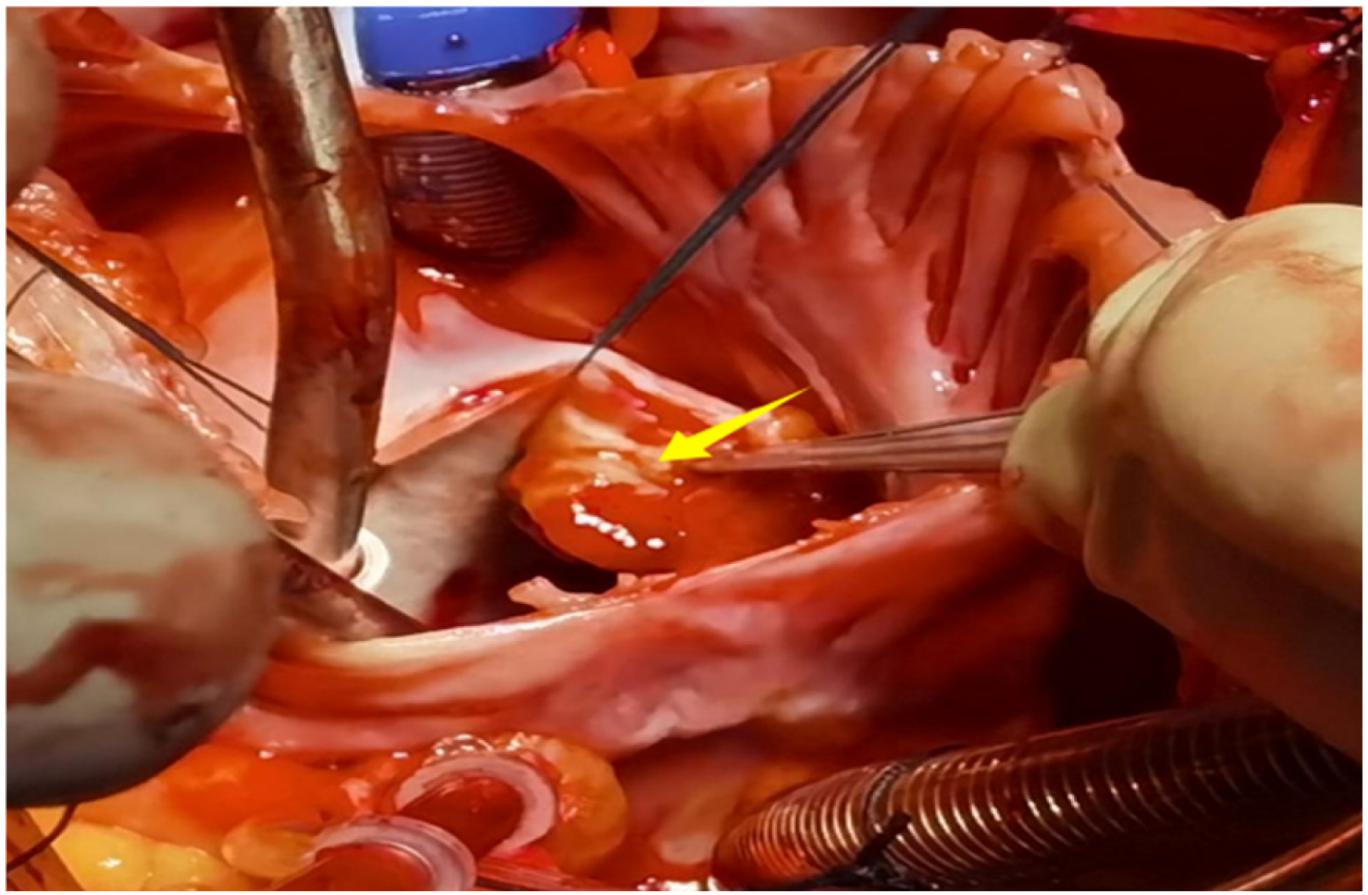
A 1.7 cm × 1.0 cm jelly-like, brittle LA mass attached to the atrial septal fossa ovale.

**Figure 8:**
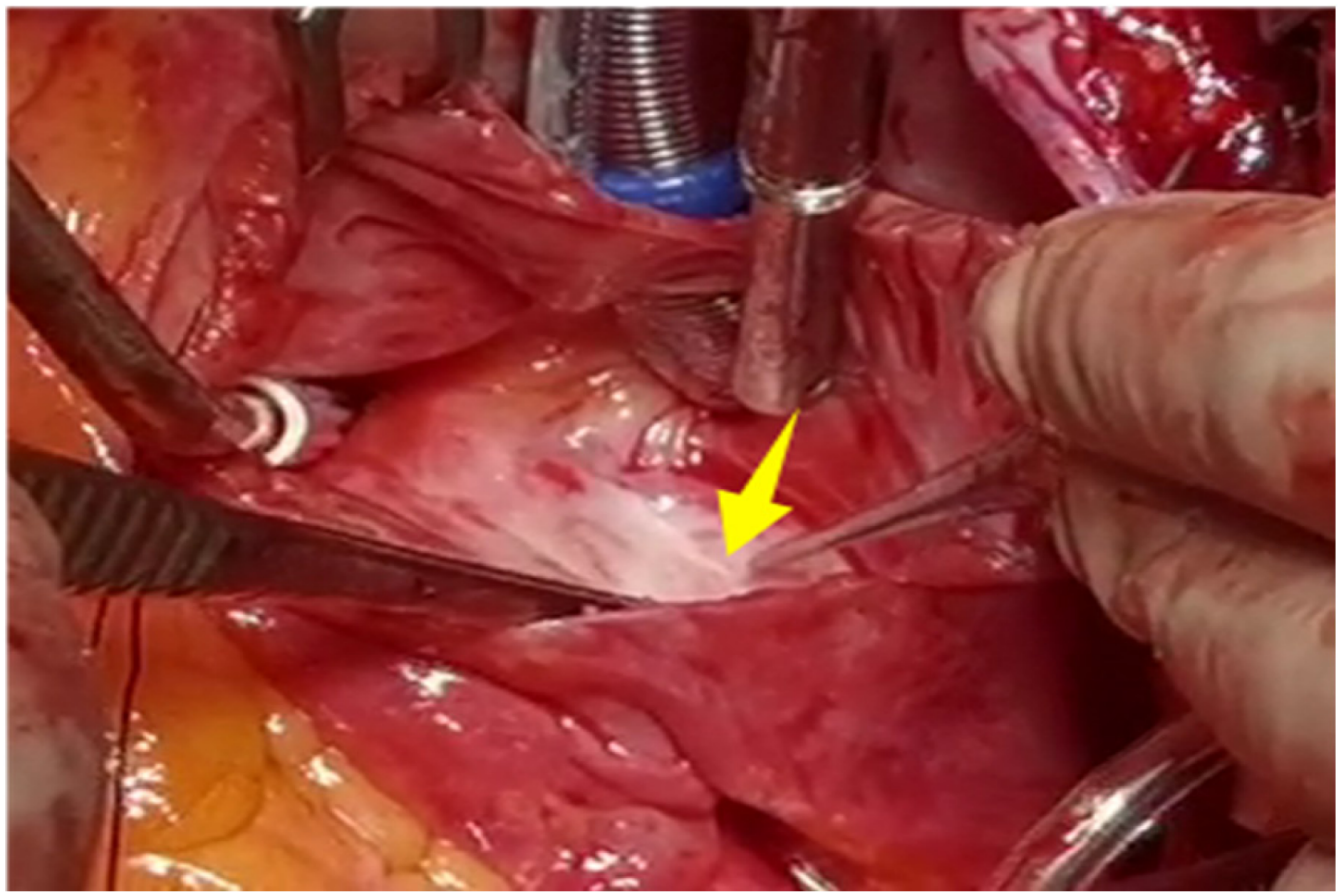
Abnormal coronary artery of the CAF was located within the atrial septal muscle bundle.

**Figure 9:**
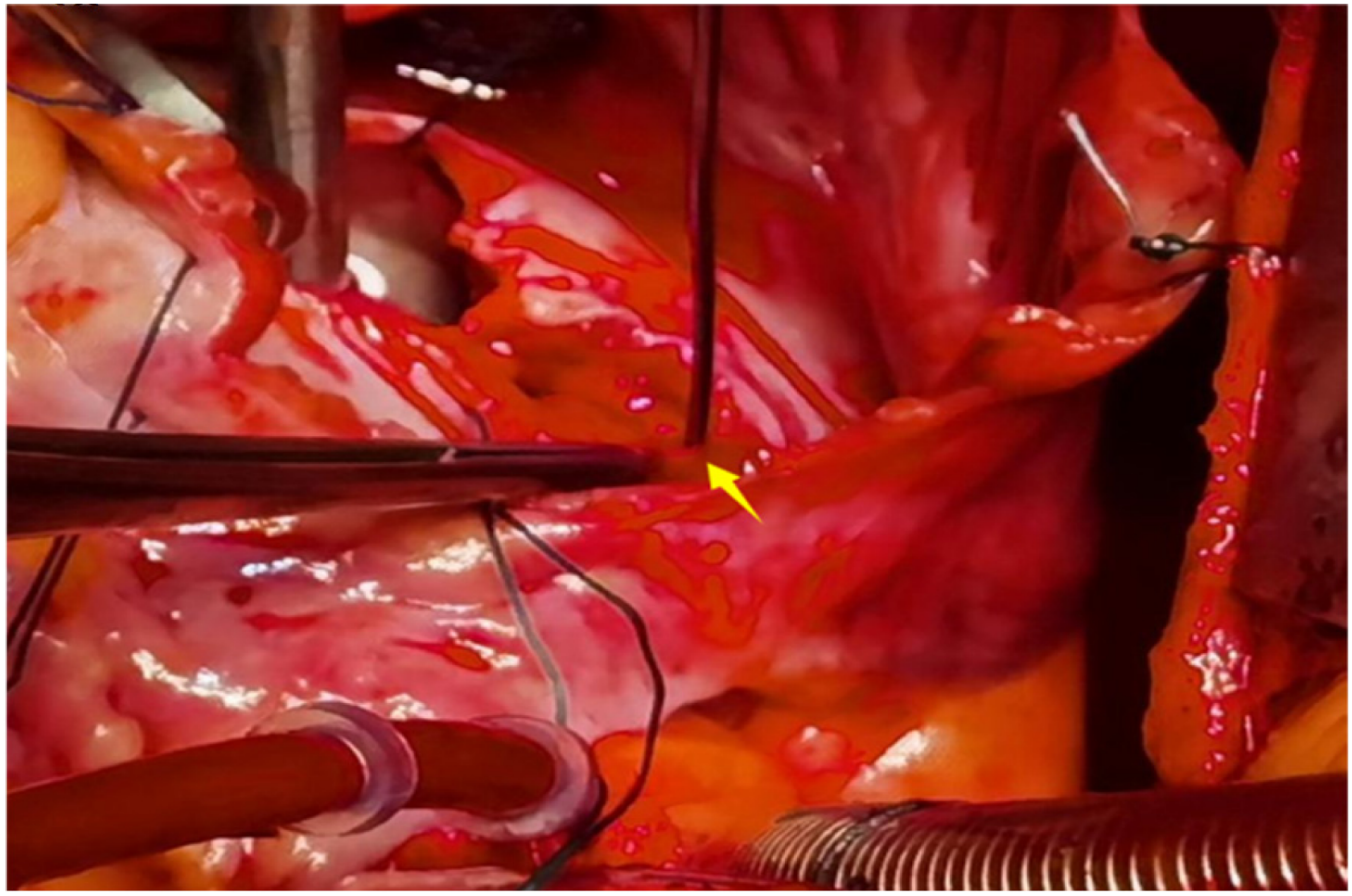
The abnormal opening of the feeding artery was located at the upper end of the atrial septal incision.

**Figure 10:**
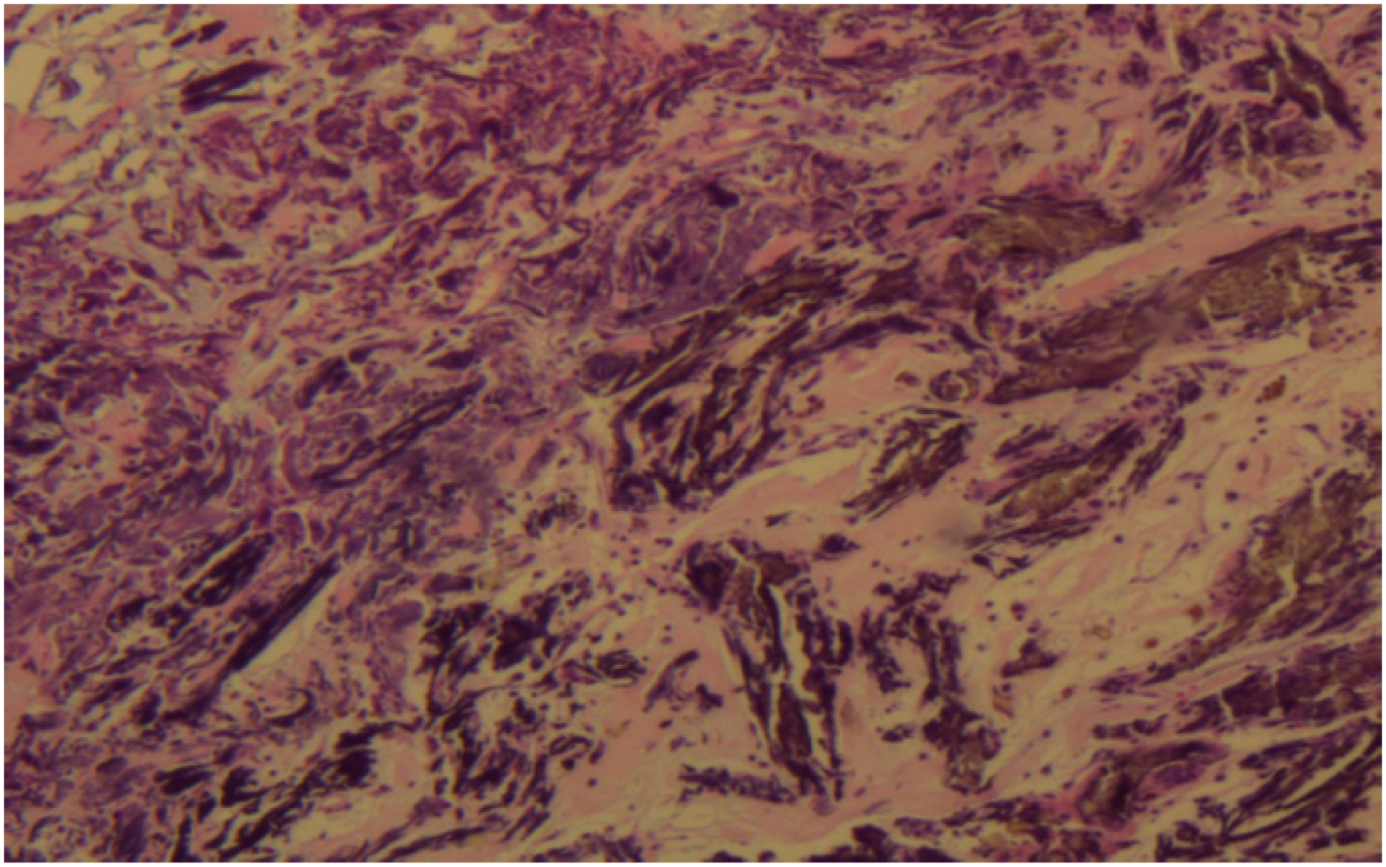
Histopathological analysis confirmed the diagnosis of myxoma.

A 56-year-old man with a 5-year history of paroxysmal palpitations, which have worsened over the past year, was diagnosed with atrial fibrillation. His vital signs and test results were within the normal range at that time. During evaluation, two- (2D) and three-dimensional (3D) transesophageal echocardiography (TEE) revealed a 17.8 mm × 11 mm LA tumoral mass, attached to the atrial septal fossa ovale (Figure 1). Multiplane TEE identified the mass as a tumor with heterogeneous echogenicity, suggesting myxoma (Figure 2). Color Doppler images showed intra-tumoral blood flow and blood stream draining from the mass (Figure 3). Left atrium computed tomography angiography (CTA) showed no signs of thrombosis but did reveal a mass in the left atrium arising from the atrial septal (Figure 4). Coronary CTA suggested intra-tumoral neovascularization with vascular channels supplied by a branch of the LCX (Figure 5). Coronary angiography (CAG) revealed a coro-cameral fistulous connection between a branch of the LCX and the LA. Furthermore, it showed the feeding arteries of the mass arising from the LCX (Figure 6). The rest of the CAG was normal. The patient was scheduled for surgical resection of the LA mass and repair of the coronary fistula. Pre-cardiopulmonary bypass (CPB) TEE demonstrated a bloodstream spurting from the tumor with a peak velocity of 70 mm/s. Intraoperative exploration revealed a 1.7 cm × 1.0 cm jelly-like, brittle LA mass attached to the atrial septal fossa ovale (Figure 7). An abnormal coronary artery of the coronary artery fistula was located within the atrial septal muscle bundle and was approximately 1.0–1.5 mm in diameter (Figure 8). The intraoperative probe confirmed that the rupture of the feeding artery or its branches resulted in a coronary artery–left atrial fistula. The abnormal opening of the feeding artery was located at the upper end of the atrial septal incision (Figure 9). The LA mass was successfully resected via the right atrial approach using a standard hypothermic CPB. Surgical ligation was executed to ensure complete sealing of the supplying coronary branch within the atrial septum. The patient was transitioned from CPB with the support of a minimal dose of dopamine. Post-procedure TEE confirmed complete resection of the LA mass and no flow across the atrial septal. The postoperative course was uneventful, and transthoracic echocardiography (TTE) showed no abnormal flow in LA. Histopathological analysis confirmed the diagnosis of myxoma accompanied by calcification and hemosiderin accumulation, revealing a multinucleated giant cell response, extensive cardiac cell degeneration, and localized infiltration of chronic inflammatory cells into the interstitium (Figure 10). The 6-month follow-up showed no recurrence of myxoma and recovery of sinus rhythm after radiofrequency ablation.

## 3 Discussion

The literature documents only a few rare cases of left circumflex artery branch-to-left atrial fistulas resulting from the rupture of a feeding artery within a left atrial myxoma. Myxomas are the most common benign primary tumors of the heart, representing 50% of such cases. They typically occur in the LA (85%), followed by the right atrium (10%) and the ventricles (5%) (5). Despite their benign nature, they require immediate surgical treatment as they carry a risk of embolic events that can be fatal. Angiographically identifiable neovascularization has been observed in 37%–56% of symptomatic cardiac myxomas, according to studies by Van Cleemput and Fueredi et al. (6–9). A small number of case series have reported the coronary steal phenomenon, which is caused by highly vascular cardiac tumors that receive their blood supply from the coronary arteries (12). In our patient, a branch of the LCX supplied blood to the myxoma. CAFs can be acquired or congenital, and they are usually considered rare coronary anomalies (10). Acquired CAFs are further categorized as spontaneous or traumatic. The most common CAFs are from the right coronary artery (RCA) (50%–55%), followed by the left coronary artery (LCA) (35%), with a small percentage (5%) arising from both coronaries. More than 90% of fistulas drain into the right heart chambers, while approximately 8% drain into the left heart chambers (11). Although systolic or diastolic murmurs are frequently observed in more than 50% of CAF patients, the patient in the present case report did not show any cardiac murmurs during physical examination, presumably due to the reduced size of the mass and the low flow rate of the arterial fistula. The combination of myxoma and CAF may promote coronary steal, although the patient was not investigated for ischemia (12). In this case, coronary fistula repair was performed simultaneously during the operation. The initial imaging modality employed to detect a cardiac mass is TTE, which provides information on the tumor's location, size, and appearance (13). Although TTE did not identify the feeding artery and fistula drainage site in our case, it could provide valuable indications of abnormal blood flow associated with the tumor (14). A subsequent CAG revealed a feeding artery originating from the coronary artery that supplies the tumor. In summary, in our case, the blood flow from the tumor manifested as an acquired CAF due to a ruptured feeding artery originating from the LCX that supplied the left atrial myxoma. The fistula was visualized as continuous flow on TEE, and CAG confirmed its course. To conclude, we report a rare case of a complicated atrial myxoma with a coro-cameral fistula arising from its feeding branch. Cardiac tumoral neovascularization may lead to the formation of CAF secondary to spontaneous vessel rupture after tumoral necrosis. Such fistulae may further complicate the course of the disease, potentially causing coronary steal and myocardial ischemia if the tumor-feeding branch is large.

## Data Availability

The original contributions presented in the study are included in the article/Supplementary Material, further inquiries can be directed to the corresponding author.
